# Fibroadenoma in women in Ghana

**DOI:** 10.4314/pamj.v2i1.51710

**Published:** 2009-07-21

**Authors:** Chhanda Bewtra

**Affiliations:** 1 Department of Pathology, Creighton University Medical Center, Omaha, NE, USA

## Abstract

**Background:**

Fibroadenoma is the commonest benign tumor of female breast. It is particularly common in young women in Africa.

**Method:**

This paper describes the clinicopathologic features of fibroadenoma of breast in African women from central Ghana and compares them to the data from African-American women.

**Results:**

Fibroadenomas constituted 47.7% of all palpable breast masses. The median age of women was 22 years (range 14-49). Almost a third of the cases occurred in teenager. The mean size of masses was 3.8 cm (range 1-9 cm), with 22.5% showing larger sizes. A total of 16.1% had multiple and/or bilateral lesions.

**Conclusion:**

Women from Central Ghana tend to have proportionately more fibroadenomas and larger (>5 cm) variants compared to published data from African-American women, however, the average age, size, multifocality and bilaterality do not differ significantly between these two groups of women.

## Background

Fibroadenoma (FA) is the most common benign tumor of the female breast, especially in the first three decades of life [1]. FAs constitute approximately one third of all benign breast lesions [2]. FAs seem to be more common in young African-American females [3, 4], however, exact figures are hard to come by. There is conflicting literature about the incidences of FAs in different races. Some studies have shown no racial differences in frequency or age distribution in black, white, or mixed populations in South Africa [1]. Another study has shown similar absence of differences amongst Hispanics, whites and American-Indian populations [1].

This study examines the clinicopathologic features of FA in Ghanaian women and compares them with those in the general population as published in the literature.

## Methods

The data were collected from the Pathology department of Komofo Anyoke Teaching Hospital (KATH) in Kumasi, Ghana, during a 2-month period. All breast specimens (diagnostic biopsies, excisions and mastectomies) obtained for pathologic examinations received in the hospital laboratory are included. All specimens were preserved in formalin. All specimens were examined by qualified physicians and the maximum dimensions were noted. Representative sections from the masses were embedded in paraffin, sectioned, stained with Hematoxylin and Eosin and examined under light microscope. Usual histological criteria [1] were applied for diagnosis.

Clinical data included age of the patients, bilaterility and multifocality of the lesions. Other data regarding pregnancy or hormone usages were not sufficient for analysis. Comparative figures in the general population (mixed, North American) and African-American (AA) women were obtained from the literature [1, 2, 3]. Because of inherent differences in data collection, no statistical analysis was applied. Concurrent malignant lesions are also described briefly, including presenting age, clinical stage, tumor type, and grade.

## Results

Out of total 65 breast specimens, 31 (48%) were FAs, 21 (32%) cancers, and 13 (20%) miscellaneous benign diseases including breast abscesses, benign fibrocystic changes, and other non-specific lesions. No tumors were found in this group. Among the benign diseases in all age groups, FA made up 70% of the lesions. Mean age of the patients with FA was 23 years, median age was 22 years, and the age range was 14-49 years. A total of 11 cases (35%) of FAs occurred in teenage groups (19 years of age and below). The tumors had the usual histology of FA. No phylloides type tumors were seen. The mean size of the FA was 3.8 cm (range 1-9 cm) with 7 cases (22.5%) measuring more than 5 cm (giant FAs). Five patients (16%) had multifocal and/or bilateral synchronous FAs. Comparative figures for the general population and AA women are given in Table 1 [1, 2, 3, 4, 5, 6, 7].

**Table 1:** Fibroadenoma in African and African-American women

	General Population* [1,2,3,5,6]	**African (Ghana)**^†^	**African-American [4,7]**
Proportion of benign breast lesions (%)	33	70	48
Peak age range (years)	20-24	16-20	16-25
Multifocality and bilaterality (%)	12-16	16	15
Mean size (range) (cm)	2.5	3.8 (1-9)	(0.5-10)
Giant FA (%) (>5cm)	2	22.5	n/a

* Predominantly North American, mixed population,

† Present study, n/a- not available

1 Harris JR, Hellman S, Henderson IC, Kinne DW. (Eds). Breast Disease, 2nd ed. Philadelphia; J.B. Lipppincott publ. 1991. pp 35–36.

2 Memon A, Parveen S, Sangrarasi AK et al. Changing pattern of benign breast lumps in young females. World J of Med Sci. 2007; 2(1); pp 21–24.

3 Page DL, Henderson TJ. Diagnostic Histopathology of the Breast. New York; Churchill Livingstone publ.1987. pp 78.

4 .Oluwole SF, Freeman HP. Analysis of benign breast lesions in blacks. Am J Surg. 1979; 137(6); pp 786–9.

5 Goehring C, Morabia A. Epidemiology of benign breast diseases, with special attention to histologic types. Epi Reviews. 1997; 19 (2); pp 310–327.

6 Park CA, David LR, Argenta LC. Breast asymmetry: presentation of a giant fibroadenoma. The breast journal 2006; 12(5); pp 451–461.

7 El-Tamer M, Song M, Wait R. Breast masses in African American teenage girls. J of Ped Surg 1999; 34(9); pp 1401–4.

During the same period, 21 (32% of all specimens) malignant tumors were received in the laboratory. Table 2 shows the comparative figures of these tumors with FAs. The mean age was 42 years (range 25-92) and the mean tumor size was 4.6 cm (range 1.5-9 cm]. Twenty out of 21 cases were infiltrating ductal carcinoma of usual type. There was one case of lobular carcinoma and two (8%] cases were grade I tumors by the Bloom-Richardson modified grading system [1]. All others were grades II and III. One case each of focal Pagetoid nipple involvement, metaplastic squamous changes and focal mucinous carcinomas were seen mixed with usual type tumors. Clinically, one case (5%) was clinically stage I (localized in breast only). All others were of higher stages with lymph node (55%), skin (33%) and chest wall (16%) involvements. There were two cases with bilateral involvement.

**Table 2:** Benign and malignant breast lesions

	**Fibroadenoma**	**Carcinoma**
Proportion of lesion (%)	31 (48)	21 (32)
Mean age (years) (range)	23 (14-49)	42 (25-92)
Mean size (cm) (range)	3.8 (1-9)	4.6 (1.5-9)

## Discussion

The etiology of FAs is not known. The stromal and epithelial cells contain estrogen and progesterone receptors [1]. Predictably, these tumors often proliferate during pregnancy and regress after menopause. However, factors like age at menarche and menopause, parity, breastfeeding, diet, and smoking do not appear to affect the incidence of FA [5]. Oral contraceptives usage before age 20 appears to increase the risk of FA [8], however, obesity seems to provide some protection [5]. Increased FAs are seen in immunosuppressed patients with cyclosporine therapies. These have been associated with Epstein Barr virus infections [8]. The stromal cells are bcl-2 positive and it has been postulated that neoplastic growth may occur through the pathway similar to solitary fibrous tumor [8]. Except for one study showing strong association of 6q deletion with FA and other lesions [9], no consistent cytogenetic or karyotypic abnormality has been associated with FA.

Because this study is a laboratory registry audit, it does not accurately reflect the incidence and prevalence of FA in the general population in Africa. However, the literature survey shows numerous reports stating an increased incidence of FAs in young African-American women [1, 3, 4], and decreased mean age, increased size of the tumors, and increased incidence of multifocal and bilateral tumors [1, 3]. Some studies have focused on African-American women [4, 7] and on women in African countries [10, 11, 12, 13]. One report from Kenya shows that FAs constitute 49% of benign breast lesions [13]. A similar study from Nigeria shows a comparable figure of 33%. One study [11] from the rainforest region in Nigeria reported an unusually high incidence of FA (94% of all benign lesions). The cause is not known and no other similar studies are reported.

Most FAs grow up to 2-3 cm and then gradually regress [3] or cease to grow. However, approximately 2% may grow larger and become “giant fibroadenoma”. The exact definition varies, but most consider FAs larger than 5 cm to be of the”giant” variant. These may reach 20 cm or more. The present study found an unusually high number of such giant FAs: 7 out of 31 (22.5%), which has not been reported by other studies and remains unexplained.

Because the aim of this paper is to study the FA, the concurrent malignant tumors are only mentioned briefly. Of interest is the presenting age of the African cancer patients. The mean age of 42 years is significantly younger than their cohorts (61 years) in the western countries, as indicated in the SEER data from the United States [13]. Further studies are in progress.

## Conclusion

For such a common tumor, relatively little is known about the etiology and risk factors of FA. It is apparent from Table 1, that the incidence of FA is higher in Africa than in western industrialized countries, and that the incidence in African-American women falls somewhere in between. This suggests some genetic and/or environmental etiology that are yet unknown and need further investigation with appropriate racial, ethnic, or geographic discriminators.

## Competing interest

The author declares no conflicts of interest.

## Authors’ contribution

**CB:** is the sole author of this paper.

## Acknowledgement

The author thanks the Pathology Staff at the K.A.T.H. hospital in Kumasi, Ghana for providing the patient data.
